# Room‐Temperature Ferromagnetism in an Iron‐Based Zeolitic Imidazolate Framework Glass

**DOI:** 10.1002/advs.202516465

**Published:** 2025-10-24

**Authors:** Chaohui Guo, Xuan Ge, Ang Qiao, Zijuan Du, Muzhi Cai, Xuefeng Wang, Haizheng Tao, Xiujian Zhao, Yuanzheng Yue

**Affiliations:** ^1^ State Key Laboratory of Silicate Materials for Architectures Wuhan University of Technology Wuhan 430070 China; ^2^ Shanghai Key Laboratory of Materials Laser Processing and Modification School of Materials Science and Engineering Shanghai Jiao Tong University Shanghai 200240 China; ^3^ Institute of Optoelectronic Materials and Devices China Jiliang University Hangzhou 310018 China; ^4^ State Key Laboratory of Spintronics, School of Electronic Science and Engineering Nanjing University Nanjing 210093 China; ^5^ Department of Chemistry and Bioscience Aalborg University Aalborg DK‐9220 Denmark

**Keywords:** metal‐organic framework glass, order‐disorder transition, room‐temperature weak ferromagnetism, structure

## Abstract

Although many metal‐organic frameworks (MOFs) display magnetic properties, it remains unclear whether intrinsic weak ferromagnetism (WFM) can occur at room temperature within these materials. Here the discovery of the WFM is reported at room temperature in an iron‐based zeolitic imidazolate framework (ZIF) glass, specifically Fe(Im)_2_, where Im is imidazolate. It is found that antiferromagnetic behavior in the crystalline Fe‐ZIF transforms into WFM upon melt‐quenching, i.e., during the transition to a structurally disordered glassy state. This magnetic transition is attributed to the enhanced exchange interactions between adjacent Fe^II^ nodes, resulting from a reduction in the Fe^II^‐Fe^II^ correlation length from 6.2 Å in the crystalline phase to 6.0 Å in the glass. ^57^Fe Mössbauer spectroscopy reveals that the order‐to‐disorder transition leads to a transition of the low‐spin‐state in Fe^II^ to the uniform high‐spin state. The modification of the coordination environment induces room‐temperature WFM. The finding opens a pathway for the application of MOF glasses in magnetic and spintronic technologies.

## Graphical abstract

Vitrification engineering is employed to induce magnetic ordering transformation from room‐temperature antiferromagnetism of crystalline Fe‐ZIF to weak ferromagnetism of its glassy counterpart. The structure‐magnetism relationships are investigated to provide a valuable guide for tuning magnetic properties and designing of MOFs magnets.

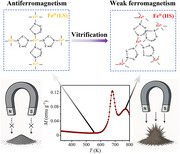

## 1 Introduction

Some metal‐organic frameworks (MOFs) have emerged as a new family of magnetic materials, showing potential for various applications, e.g., in multi‐state memory devices, spintronic systems, and magnetic sensing technologies.^[^ ^1^, ^2^, ^3^, ^4^ ^]^ However, intrinsic weak ferromagnetism (WFM), an intrinsic property observed in antiferromagnets with a nonzero magnetic moment,^[^ ^5^, ^6^ ^]^ can be observed only far below room temperature due to the weak magnetic exchange interactions in MOFs.^[^ ^7^, ^8^ ^]^ To enhance magnetic exchange interaction, the following two strategies were often employed: i) designing MOFs with proper organic ligands and metal nodes to optimize exchange pathways,^[^ ^9^ ^]^ and ii) employing external stimuli such as temperature, pressure, or guest molecule sorption.^[^ ^10^, ^11^, ^12^ ^]^ Significant efforts have been made to achieve WFM in crystalline MOFs. Li et al. synthesized a 3D MOF with well‐separated diamagnetic [V_4_O_12_]^4−^ cluster units and semi‐rigid ligands, which exhibited WFM below 3.6 K.^[^ ^13^ ^]^ Rubio‐Giménez et al. observed modulation of spontaneous magnetization between 10 and 16 K by incorporating different amounts of Fe(III) into NiFe‐MOF‐74.^[^ ^14^ ^]^ By creating cobalt quantum dots in the Cu‐MOF‐74, Mao et al. were able to enhance the magnetic ordering transition temperature from 7.8 to 275 K at 1 000 Oe.^[^ ^15^ ^]^ Feng et al. employed a ligand‐cleavage strategy to achieve room‐temperature ferromagnetism in Cu‐MOF.^[^ ^7^ ^]^ By using a 2D Ising model, Li et al. predicted that NiMn‐OIPc possesses a ferromagnetic ordering temperature exceeding 600 K.^[^ ^16^ ^]^ Nevertheless, no method has yet been established to achieve room‐temperature intrinsic WFM in MOFs.

In the present work, we took a different strategy, i.e., vitrification of MOFs as it can effectively regulate their framework and microscopic structure.^[^ ^17^, ^18^, ^19^ ^]^ Upon glass formation, the pore structure of MOFs is significantly distorted and even partially collapsed, while the structural order is lost at different length‐scales.^[^ ^20^ ^]^ Consequently, the interactions between metal nodes and organic ligands are altered, thereby influencing the MOFs’ functionalities such as gas‐separation, optical and electrochemical properties.^[^ ^21^, ^22^, ^23^, ^24^ ^]^ It is known that weak ferromagnetic ordering in MOFs is sensitive to the change of their structure.^[^ ^25^ ^]^ It is also known that vitrification leads to structural disordering, coordination symmetry breaking, as well as the electronic configuration changing.^[^ ^26^, ^27^, ^28^, ^29^, ^30^ ^]^ Thus, vitrification is expected to impact magnetic ordering in MOFs. For instance, Fe‐^t^Bubipy glass exhibited a WFM below 14 K, i.e., a certain degree of antiferromagnetic ordering with a small remanent magnetization caused by spin canting.^[^ ^31^ ^]^ A similar phenomenon was observed in MUV‐29 glass, which exhibited a WFM below 15 K.^[^ ^32^ ^]^ However, to date, no room‐temperature intrinsic WFM has been reported in MOFs.

Iron nodes in ZIFs have various coordination numbers to organic ligands, thereby providing enhanced flexibility and tunability in both structure and properties.^[^ ^33^ ^]^ In this connection, an iron‐based ZIF (Fe‐ZIF) was selected for the present study. We employed the melt‐quenching approach to prepare a Fe‐ZIF glass from its crystalline counterpart. We observed intrinsic WFM in a Fe‐ZIF glass at room temperature. The magnetic transition caused by vitrification was detected through both field‐dependent magnetization (*M*‐*H*) and temperature‐dependent magnetization (*M‐T*) measurements. Both pair‐distribution functions (PDF) and ^57^Fe Mössbauer spectroscopy were employed to uncover the microscopic origin of the magnetic transition.

## 2 Results and Discussion

### 2.1 Phase Transitions and Glass Formation

**Figure 1:**
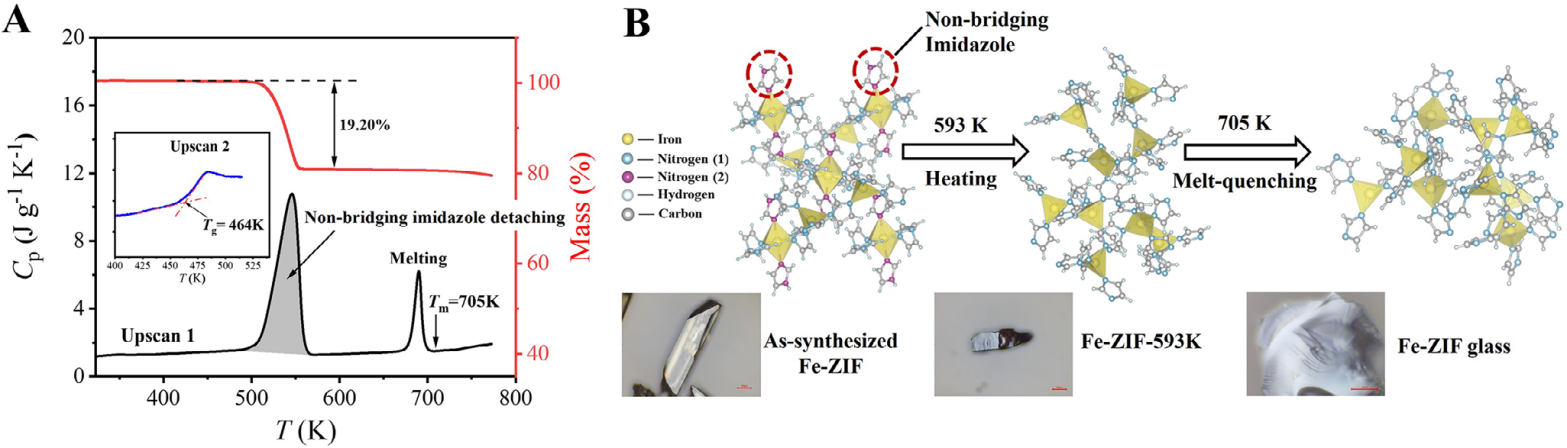
Phase transitions and glass formation in an iron‐based zeolitic imidazolate framework. A) Thermogravimetry (mass %) (red) and isobaric heat capacity (*C*_p_) (black) curves of the as‐synthesized Fe‐ZIF crystal at the upscan rate of 10 K min^−1^. Inset: the glass transition in Fe‐ZIF glass (*T*_g_ = 464 K). B) Schematic representation of structural evolution in Fe‐ZIF during heating and melt‐quenching (upper panel), along with optical images of the as‐synthesized crystalline sample, the sample heated to 593 K, and the melt‐quenched product (lower panel). The atomic configuration of the Fe‐ZIF unit cell is adopted from ref. [34]

The Fe‐ZIF samples are first synthesized through a solvent‐free approach and then characterized by X‐ray diffraction (XRD), scanning electron microscopy (SEM), differential scanning calorimetry (DSC), and thermogravimetry (TG). The XRD patterns (Figure S1, Supporting Information) confirm the crystalline phase of the samples belongs to the monoclinic space group *P*2_1_/*c*.^[^ ^34^ ^]^ SEM images (Figure S2, Supporting Information) reveal that the samples consist of uniformly rectangular particles with an average size of ≈900 *µm* in lateral dimension. The isobaric heat capacity (*C*_p_) curve derived from the first DSC upscan (**Figure** **1**A) exhibits two endothermic peaks. The first one in the range of 480–571 K is attributed to the detachment of non‐bridging imidazole molecules from the framework (Figure S3, Supporting Information), which is reflected by a mass loss of 19.2%, as shown in the TG curve. The second peak is ascribed to the melting process with a liquidus temperature (*T*_m_) of 705 K. Upon melt‐quenching, Fe‐ZIF glass forms, as evidenced by the glass transition peak featured by the onset of temperature (*T_g_*) of 464 K during the second upscan (inset of Figure 1A). Its amorphous nature is confirmed by the absence of sharp crystalline peaks in the XRD pattern (Figure S1, Supporting Information). The micro‐optical images (Figure 1B; Figure S2, Supporting Information) demonstrate the typical fracture pattern characteristic of inorganic bulk glasses.^[^ ^35^ ^]^

The as‐synthesized Fe‐ZIF crystal features a three‐dimensional framework comprising [FeIm_4_] tetrahedral units and [FeIm_6_] octahedral units bridged by imidazolate ligands through coordination bonds (Figure 1B). Notably, two non‐bridging imidazole ligands in each [FeIm_6_] octahedral unit detach during heating up to 571 K (Figure 1A). This induces a phase transition from the as‐synthesized Fe‐ZIF crystalline phase to another crystalline phase (designated as Fe‐ZIF‐593 K, Figure S1, Supporting Information), involving corner‐shared [FeIm_4_] units. However, the morphologies of as‐synthesized Fe‐ZIF and Fe‐ZIF‐593 K samples remain similar (Figure 1B; Figure S2, Supporting Information).

### 2.2 Magnetic Response

**Figure 2:**
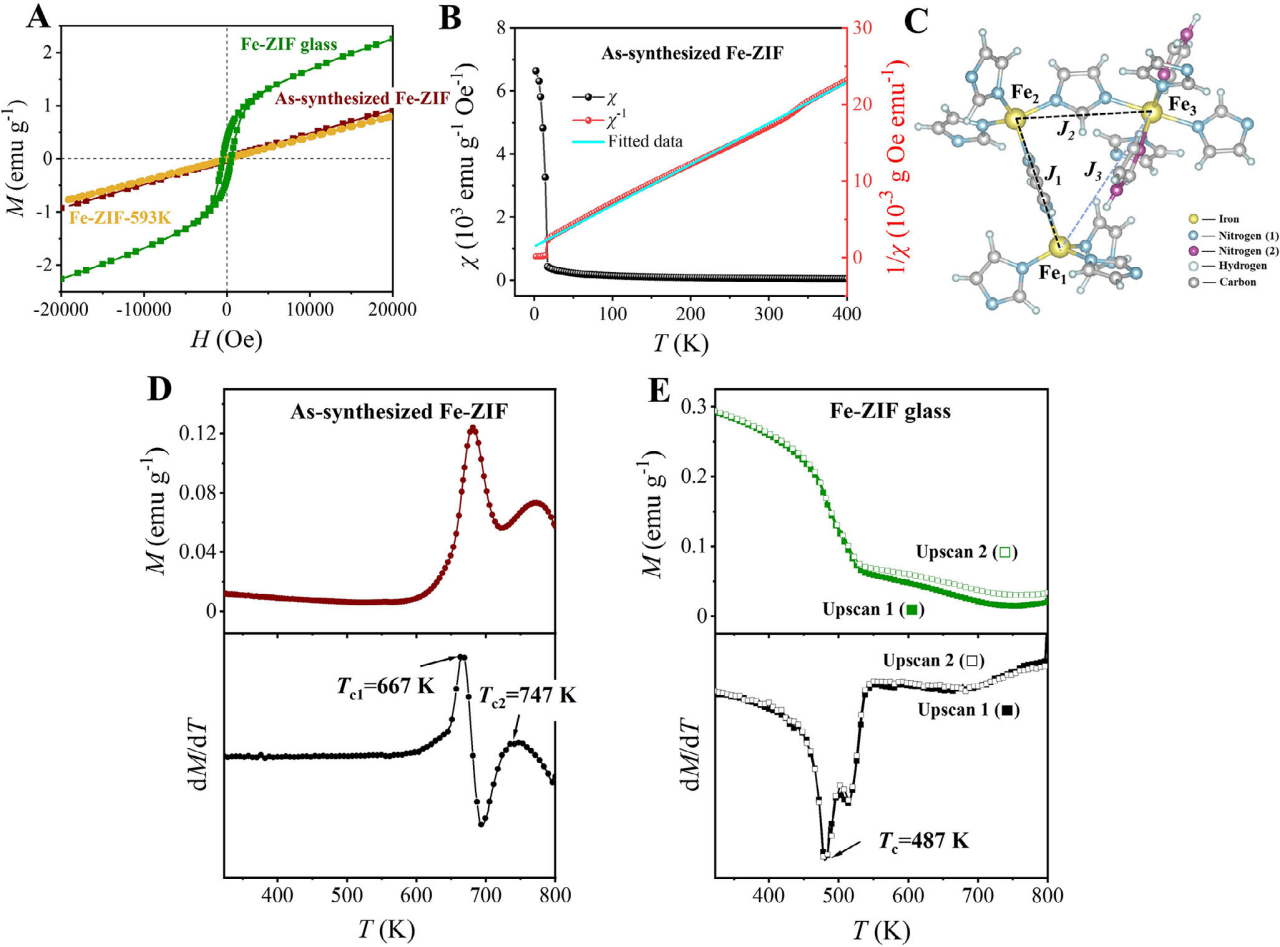
Characterizations of magnetic properties. A) Field‐dependent magnetization (*M*‐*H*) curves of as‐synthesized Fe‐ZIF, Fe‐ZIF‐593 K, and Fe‐ZIF glass, recorded at 300 K up to ± 20 000 Oe. B) Temperature dependence of magnetic susceptibility (*χ‐T*) and its reciprocal (*χ* ^−1^ *‐T*) curves of Fe‐ZIF. Light blue solid line: the fits of *χ* ^−1^ *‐T* data to the Curie‐Weiss equation. C) Schematic illustration of magnetic exchange interactions in Fe‐ZIF: *J*_1_ (Fe_1_ – Fe_2_), *J*_2_ (Fe_2_ – Fe_3_), *J*_3_ (Fe_3_ – Fe_1_). (D‐E) Temperature‐dependent magnetization (*M‐T*) and their first derivatives (d*M*/d*T*) curves for both the as‐synthesized Fe‐ZIF crystal and the Fe‐ZIF glass, respectively.

To probe the evolution of magnetic properties in the as‐synthesized Fe‐ZIF crystal upon vitrification, comparative *M*‐*H* and *M‐T* measurements were performed on both the crystalline and glassy Fe‐ZIF samples. For the as‐synthesized Fe‐ZIF and Fe‐ZIF‐593 K crystals, the *M* value linearly increases with *H* value (**Figure** **2**A), implying no magnetic hysteresis and nearly zero coercivity. In contrast, the Fe‐ZIF glass exhibits pronounced magnetic hysteresis, characterized by a coercive field (*H*_c_ = 510 Oe) and remanent magnetization (*M*_r_ = 0.38 emu g^−1^), as shown in Figure 2A. Remarkably, the magnetization of Fe‐ZIF glass remains unsaturated even up to ± 20 000 Oe.

Furthermore, the temperature‐dependent magnetic susceptibility can be determined through both the reciprocal susceptibility (*χ* ^−1^‐*T*) and the direct susceptibility (*χ‐T*) as functions of temperature (Figure 2B). Both values were derived from *M*‐*T* measurements of as‐synthesized Fe‐ZIF (Figure S4, Supporting Information). According to the Curie‐Weiss law, magnetic susceptibility is defined as *χ* = *C/*(*T*‐*θ*), where *C* is the Curie constant and *θ* is the Curie‐Weiss temperature. Depending on the type of coupling between magnetic ions, the value of *θ* can be either negative (antiferromagnetic interactions) or positive (ferromagnetic interactions).^[^ ^7^ ^]^ The *θ* value of the as‐synthesized Fe‐ZIF crystal is calculated as −27.02 K by fitting the *χ* ^−1^‐*T* correlation to the Curie‐Weiss law (light blue line in Figure 2B) over the temperature range of 2–400 K. This confirms the presence of antiferromagnetic interactions in this temperature range.

To gain insights into the magnetic behavior of the as‐synthesized Fe‐ZIF crystal, its magnetic ground state is calculated using the density functional theory (DFT) by incorporating appropriate U‐values (see the details in Supporting Information). Based on the Heisenberg model $H=-J\sum_{\left\langle \mathrm{ij}\right\rangle }\vec{S_{i}}\cdot \vec{S_{j}}$, where $\vec{S_{i}}$ and $\vec{S_{i}}$ represent the spin operators of magnetic moments at neighboring sites *i* and *j*, respectively, and *J* is the exchange constant, the values of *J*_1_, *J*_2,_ and *J*_3_ are computed to be −1.5334, −0.4040, and −0.0071 meV, respectively. These calculations take into account both first‐nearest and second‐nearest neighbors, as illustrated by the Fe_3_(Im)_6_(Him)_2_ cluster in Figure 2C.^[^ ^36^ ^]^ These results indicate the presence of the antiferromagnetic coupling among Fe_1_, Fe_2,_ and Fe_3_.^[^ ^37^ ^]^ Notably, the iron‐based MOFs exhibit magnetic exchange interactions between neighboring iron nodes through the bridging ligands.^[^ ^38^ ^]^ There is no imidazole ligand that directly bridges Fe_3_ and Fe_1_. Moreover, the distance between Fe_3_ and Fe_1_ atoms exceeds 7 Å (Table S1, Supporting Information), which is too far for direct interaction.^[^ ^39^ ^]^ Thus, the calculated magnetic exchange constant *J*_3_ between Fe_3_ and Fe_1_ should not be considered. Therefore, the antiferromagnetic behavior in the as‐synthesized Fe‐ZIF should be attributed to the exchange interactions of both Fe_1_‐Fe_2_ and Fe_2_‐Fe_3_ pairs, which are mediated by the imidazolate ligand.^[^ ^40^ ^]^

Compared to the as‐synthesized Fe‐ZIF, the Fe‐ZIF‐593 K sample exhibits a similar *χ‐T* curve (Figure S5, Supporting Information), i.e., the *χ* value gradually increases from near zero as the temperature decreases from 400 to 2 K. Additionally, no significant change in magnetization is observed between 323 and 593 K in the *M*‐*T* curve, indicating that Fe‐ZIF‐593 K and the as‐synthesized Fe‐ZIF possess similar magnetic characteristics, namely antiferromagnetism (AFM). In contrast, Fe‐ZIF glass exhibits a clearly non‐zero *χ* value at 400 K (Figure S6, Supporting Information). The presence of a non‐zero *χ* and well‐defined hysteresis loop, along with the absence of saturation in magnetization at high magnetic fields, provides compelling evidence for the WFM behavior of the Fe‐ZIF glass.^[^ ^41^, ^42^ ^]^ The non‐zero magnetization observed in the *M‐T* curves also confirms the occurrence of the WFM in the Fe‐ZIF glass at room temperature (Figure 2E). Furthermore, the corresponding d*M*/d*T* curve of Fe‐ZIF glass exhibits a single peak, accompanied by a rapid decrease in magnetization. This implies that there is a single critical temperature (*T*_c_) of 487 K in Fe‐ZIF glass, where a weak ferromagnetic state transitions to a paramagnetic state. This magnetic state transition is reversible in the Fe‐ZIF glass.

Moreover, zero‐field‐cooled (ZFC) and field‐cooled (FC) measurements were performed to provide deeper insight into the temperature‐dependent magnetic behavior of the Fe‐ZIF glass. As shown in Figure S7 (Supporting Information), a distinct bifurcation between the ZFC and FC curves was observed below 400 K, indicative of cluster blocking and the pinning of magnetic moments at narrow domain walls.^[^ ^43^, ^44^ ^]^ Notably, this bifurcation persists above 400 K. Such a divergence between ZFC and FC data is characteristic of spin‐glass‐like freezing, confirming the presence of spin‐glass behavior in the Fe‐ZIF glass.^[^ ^45^, ^46^ ^]^

### 2.3 Mechanisms of Room‐Temperature WFM in Fe‐ZIF Glass

After the determination of the magnetic properties of both the Fe‐ZIF crystal and Fe‐ZIF glass, the immediate question arises: what is the structural origin of the transition from AFM to WFM upon vitrification? To answer this question, we employed in situ magnetic and structural characterizations. From the *M*‐*T* and d*M*/d*T* curves of the Fe‐ZIF crystal in the temperature range of 323–800 K (Figure 2D), we find its critical temperatures of magnetic ordering. Evidently, the magnetization value drastically varies within the same temperature range as that of the crystal melting.

**Figure 3:**
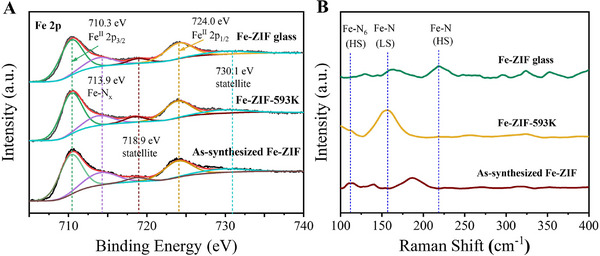
Structural characterizations. A) High resolution of Fe 2p XPS spectra of Fe‐ZIF, Fe‐ZIF‐593 K, and Fe‐ZIF glass. B) Normalized FT‐Raman spectra of Fe‐ZIF, Fe‐ZIF‐593 K, and Fe‐ZIF glass, recorded at the region of 100–400 cm^−1^. HS: high spin; LS: low spin.

The magnetic properties of MOFs are governed by the magnetic exchange interactions between spin carriers mediated by ligands, i.e., by the magnitudes and relative orientations of spin carriers.^[^ ^47^, ^48^, ^49^ ^]^ The magnitudes of spin carriers in MOFs are mainly affected by the valence and spin states.^[^ ^50^ ^]^ As shown by the X‐ray photoelectron spectra (XPS) in **Figures** **3**A and S8 (Supporting Information), for all three samples, the peaks of the Fe 2p_1/2_ and Fe 2p_3/2_ orbitals appear around 710.3 and 723.9 eV respectively, along with satellite peaks at ≈718.7 and 729.6 eV. These features are consistent with those reported in the MOF materials with +2 valence Fe ions as metal nodes,^[^ ^51^, ^52^ ^]^ indicating that the evolution of magnetization with field and temperature (Figure 2A,D) cannot be ascribed to the change in the valence state of Fe nodes.

**Figure 4:**
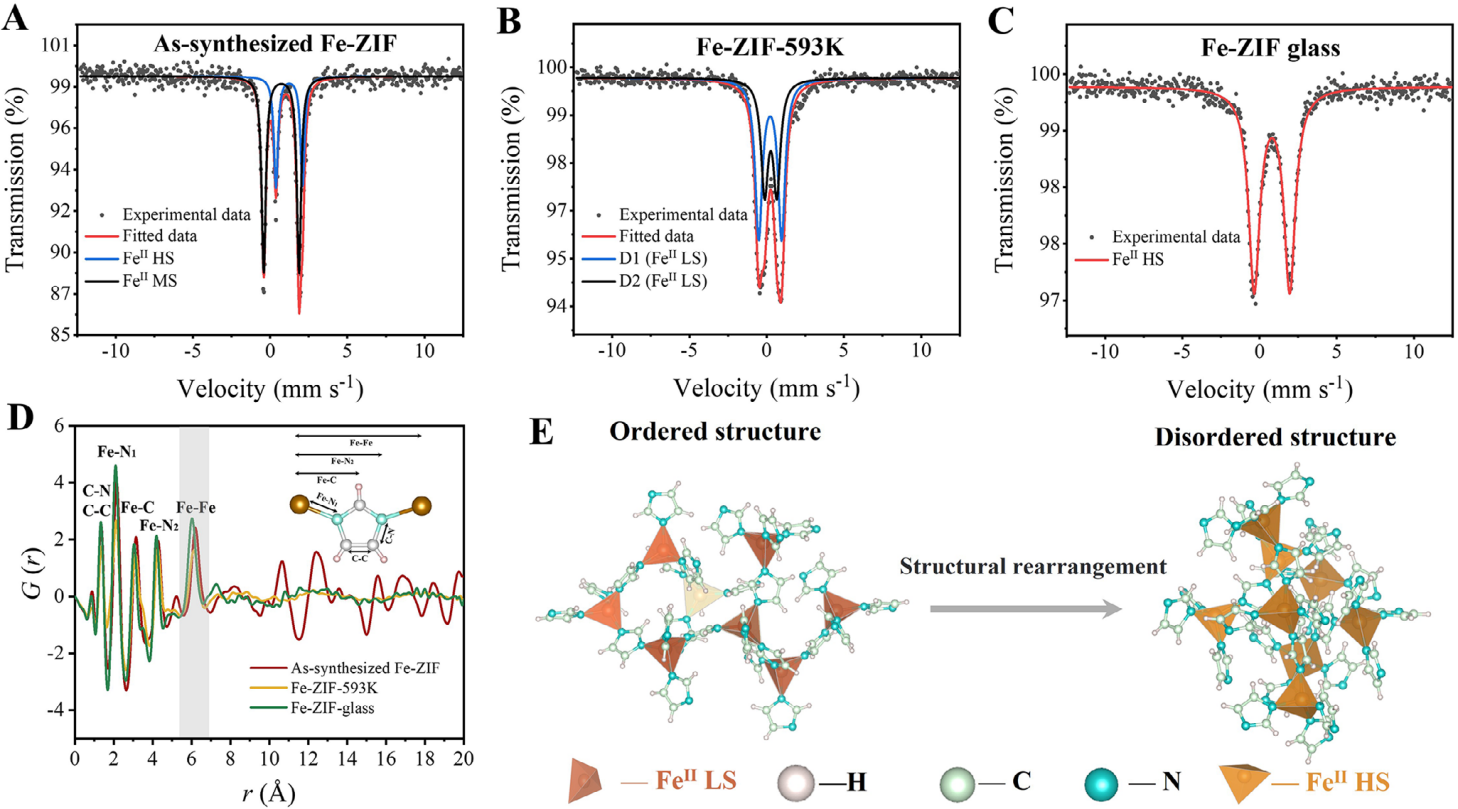
Spin interactions and structural evolution. A–C) ^57^Fe Mössbauer transmission spectra of Fe‐ZIF, Fe‐ZIF‐593 K, and Fe‐ZIF glass. D) X‐ray pair distribution functions *G*(*r*). E) Schematic diagram of the structural order‐disorder transition and the spin state transition of metal nodes upon vitrification.

The electronic spin state of the metal nodes can affect the metal‐ligand bonds, which can be monitored by Raman spectroscopy.^[^ ^53^ ^]^ As the Raman spectra shown in Figure 3B, the peak at 108 cm^−1^ of Fe‐ZIF is related to Fe‐N high‐spin (HS) state in octahedral [FeIm_6_] units,^[^ ^54^ ^]^ whereas those at 158 and 220 cm^−1^ are associated with Fe‐N low‐spin (LS) in Fe‐ZIF‐593 K^[^ ^55^ ^]^ and HS states in Fe‐ZIF glass,^[^ ^56^, ^57^ ^]^ respectively. To further explore the changes in spin state, the ^57^Fe Mössbauer measurements were performed (**Figure** **4**A–C). The changes in ^57^Fe Mössbauer parameters, namely the isomer shift (IS) and quadrupole splitting (QS), upon heating indicate significant variations in the electron density and coordination environment of the Fe nodes associated with structural ordering. The IS reflects the density of s‐electrons at the absorbing Fe nucleus,^[^ ^58^ ^]^ whereas the QS is governed by the local chemical environment of the absorbing Fe nucleus and the occupation of the 3d orbitals.^[^ ^59^ ^]^ Generally, the IS value of Fe^II^ in the HS state exceeds 0.8 mm s^−1^, while that of Fe^II^ in the LS state is typically below 0.4 mm s^−1^.^[^ ^60^ ^]^ As shown in Figure 4A, the spectrum of the as‐synthesized Fe‐ZIF is deconvoluted into two doublets with parameters of IS = 1.24 mm s^−1^, QS = 1.72 mm s^−1^ and IS = 0.72 mm s^−1^, and QS = 2.29 mm s^−1^. The observed IS of 0.72 mm s^−1^ falls between the HS and LS ranges, which has been reported in the literature as indicative of a medium‐spin (MS) state of FeN_4_ centers.^[^ ^61^, ^62^ ^]^ Therefore, the two doublets are reasonably assigned to Fe^II^ in the HS and MS state. In contrast, the two new doublets of Fe‐ZIF‐593 K (D1, IS = 0.22 mm s^−1^, QS = 1.48 mm s^−1^; D2, IS = 0.26 mm s^−1^, QS = 0.79 mm s^−1^) are assigned to Fe^II^ LS state (Figure 4B).^[^ ^63^, ^64^ ^]^ This striking difference between the two samples implies that the detaching process of non‐bridging imidazole molecules causes an alteration in spin state, but no change in antiferromagnetic ordering. However, upon vitrification, the D1 and D2 peaks become a single doublet related to Fe^II^ HS (IS = 0.8 mm s^−1^, QS = 2.30 mm s^−1^), as displayed in Figure 4C, indicating that melt‐quenching induces the LS to HS transition.^[^ ^65^ ^]^ Such a transition has also been observed in a [Fe^II^‐NC‐W^v^]‐based coordination polymer, attributed to light‐excitation. But the switch of magnetic hysteresis in this polymer occurred below 3.3 K.^[^ ^66^ ^]^ Typically, the cooperativity of electron spins is insufficient to induce room‐temperature weak ferromagnetic behavior in MOFs.

To further clarify the mechanism of the alteration in magnetism, the structures of the three studied samples were probed by high‐energy synchrotron X‐ray diffraction. According to the reduced pair distribution function *G*(*r*) (Figure 4D), specific interatomic interactions can be assigned to these peaks: C─C/C─N (1.34 Å), Fe─N_1_ (2.08 Å), Fe─C (3.16 Å), Fe─N_2_ (4.30 Å), and Fe─Fe (6.20 Å). Upon vitrification, a significant decrease in the Fe─Fe distance from 6.20 Å in both Fe‐ZIF and Fe‐ZIF‐593 K to 6.00 Å in Fe‐ZIF glass is detected, indicating a striking short‐range structural evolution in Fe‐ZIF network. The shortening of this bond length reflects a substantial distortion in the structural units ([FeIm_4_] tetrahedra), which could fundamentally be attributed to the unique short‐range disorder characteristic in Zn‐ZIF glass evidenced by ultrahigh‐field ^67^Zn NMR.^[^ ^20^ ^]^

From the above‐derived information on structure and electronic configuration, we infer that vitrification modifies the local coordination environment of Fe^II^ nodes, particularly increases the degree of the structural disorder at the short‐range scale, i.e., within and/or between neighboring [FeIm_4_] tetrahedral units. In other words, the melt‐quenching process alters both the coordination bond length of Fe─N and the angle distribution of N─Fe─N. This alteration leads to a distortion of electronic conjugation and hence to an LS‐to‐HS transition. Moreover, vitrification may induce an expansion of the electron cloud, thereby facilitating the orbital hybridization between d orbitals of Fe^II^ nodes and *π* orbital of imidazole. This phenomenon may enhance the d‐*π*‐d exchange interaction between Fe^II^ nodes bridged by imidazole, ultimately resulting in a significant enhancement of magnetic exchange interactions.^[^ ^67^, ^68^ ^]^ The above‐proposed mechanisms are schematically illustrated in Figure 4E, where Fe^II^ nodes and imidazole ligands in Fe‐ZIF‐593 K are arranged in a well‐defined ordered structure. Their Fe^II^ nodes adopt an antiparallel configuration, where exchange interactions occur through bridging the imidazole ligands, leading to antiferromagnetic coupling. Upon vitrification, both the internal structure of tetrahedral [FeIm_4_] units and the relative orientations of Fe^II^ nodes along the chains progressively evolves toward disorder. The modification of the coordination environment, coupled with symmetry‐breaking structural frustration, results in an asymmetric exchange coupling between spins. Such structural evolution activates the Dzyaloshinskii‐Moriya interactions and thereby stabilizes weak ferromagnetic structures. These factors synergistically promote the emergence of room‐temperature WFM in Fe‐ZIF glass. In addition, vitrification, involving the dissociation and reformation of coordination bonds between metal nodes and organic ligands, may regulate both spin states of the carriers and the exchange interactions mediated by bridging ligands.

## 3 Conclusion

We directly observed the magnetic ordering transformation from room‐temperature antiferromagnetism of crystalline Fe‐ZIF to WFM of its glassy counterpart. This notable transition, induced by melt‐quenching, highlights the potential of controlled vitrification as a strategy for tuning the magnetic properties of MOFs. Specifically, systematic modulation of quenching rates and ligand chemistry offers a pathway to engineer magnetic exchange interactions, particularly in the role of Dzyaloshinskii‐Moriya interactions of MOF glasses. Ultimately, our work provides a valuable guide for the design of MOFs magnets for magneto‐optical sensors and energy‐efficient magnetic switches.

## Conflict of Interest

The authors declare no conflict of interest.

## Supporting information



Supporting Information

## Data Availability

The data that support the findings of this study are available in the supplementary material of this article.
