# Supplementary material for: Room‐Temperature Ferromagnetism in an Iron‐Based Zeolitic Imidazolate Framework Glass
Source: Adv Sci (Weinh). 2025 Oct 24;13(3):e16465. doi: 10.1002/advs.202516465 (PMC12806550; doi:10.1002/advs.202516465)
Supplement: Supplementary file 1 — Supporting Information [file ADVS-13-e16465-s001.pdf]

Supporting Information

Room-temperature ferromagnetism in an iron-based zeolitic imidazolate framework glass

Chaohui Guo[1,+], Xuan Ge[2,+], Ang Qiao[1,*], Zijuan Du[1], Muzhi Cai[3], Xuefeng Wang[4], Haizheng Tao[1,*], Xiujian Zhao[1], Yuanzheng Yue[5,*]

[1] C. Guo, A. Qiao, Z. Du, H. Tao, X. Zhao

State Key Laboratory of Silicate Materials for Architectures, Wuhan University of Technology, Wuhan 430070, China

E-mail: qiaoang@whut.edu.cn; thz@whut.edu.cn

[2] X. Ge

Shanghai Key Laboratory of Materials Laser Processing and Modification, School of Materials Science and Engineering, Shanghai Jiao Tong University, Shanghai 200240, China

[3] M. Cai

Institute of Optoelectronic Materials and Devices, China Jiliang University, Hangzhou 310018, China

[4] X. Wang

State Key Laboratory of Spintronics, School of Electronic Science and Engineering, Nanjing University, Nanjing 210093, China

[5] Y. Yue

Department of Chemistry and Bioscience, Aalborg University, DK-9220 Aalborg, Denmark

E-mail: yy@bio.aau.dk

[+] These authors contributed equally: Chaohui Guo, Xuan Ge

Table of Contents

Materials and methods **3**

Synthesis of as-synthesized Fe-ZIF 3

Synthesis of Fe-ZIF-593 K and Fe-ZIF glass 3

Calorimetric analysis 3

Measurements of magnetic properties 3

X-ray diffraction 4

Scanning electron microscope 4

Optical microscope 4

Differential scanning calorimetry - Thermogravimetric analysis - Mass spectrometry 4

FT-Raman spectroscopy 4

Mössbauer spectroscopy 4

X-ray photoelectron spectroscopy 5

High-energy synchrotron X-ray diffraction 5

Calculation Details 6

Supplementary Figures **7**

Figure S1. XRD patterns recorded at room temperature for as-synthesized Fe-ZIF, Fe-ZIF-593K and Fe-ZIF glass. 7

Figure S2. SEM images of the as-synthesized Fe-ZIF (A-B), Fe-ZIF-593K (C-D), and Fe-ZIF glass (E-F). 8

Figure S3. TG-DSC-MS study of as-synthesized Fe-ZIF. 9

Figure S4. Magnetization versus temperature (*M-T*) curve of as-synthesized Fe-ZIF. 10

Figure S5. Temperature dependence of magnetic susceptibility (χ-T) curves of Fe-ZIF-593K 11

Figure S6. Temperature dependence of magnetic susceptibility (χ-T) curves of Fe-ZIF glass 12

Figure S7. Zero-field cooled (ZFC) and field cooled (FC) curves of Fe-ZIF glass 13

Figure S8. Survey XPS spectra of as-synthesized Fe-ZIF, Fe-ZIF-593K and Fe-ZIF glass. 14

Figure S9. Normalized FT-Raman spectra recorded at the region of 100-2000 cm^{-1} of as-synthesized Fe-ZIF, Fe-ZIF-593 K and Fe-ZIF glass. 15

Supplementary Tables **16**

Table S1. Calculated Magnetic moments, exchange coupling constants and their spin-interactions in the as-synthesized Fe-ZIF. 16

Table S2. Raman peaks assignments. 17

Table S3. Mössbauer parameters derived from the fittings. 18

References **19**

Materials and methods

Synthesis of as-synthesized Fe-ZIF

The as-synthesized Fe-ZIF crystals were prepared by using a solvent-free synthesis method. In detail, 0.01 mol ferrocene and 0.02 mol imidazole were mixed under Ar atmosphere and put into a 10 ml Teflon-lined autoclave, and then the autoclave was tightly sealed by a stainless outer shell. Subsequently, the reactor was placed in an oven at 423 K for 96 hours and then cooling down to room temperature. The products were washed by N,N-dimethylformamide for five times, and dried at 353 K for 24 hours in a vacuum oven. Finally, the yellow powder was acquired, designated as as-synthesized Fe-ZIF.

Synthesis of Fe-ZIF-593 K and Fe-ZIF glass

The Fe-ZIF-593K sample was obtained by dynamically heating the as-synthesized Fe-ZIF sample to 593 K under an Ar atmosphere in the differential scanning calorimeter (DSC) at a heating rate of 10 K min^{-1}. Similarly, the Fe-ZIF glass sample was prepared by melt-quenching the as-synthesized Fe-ZIF sample, i.e., heating the as-synthesized Fe-ZIF to the temperature (773 K) that is above the melting point (705 K) under argon in the DSC at 10 K min^{-1} and then cooling to room temperature.

Calorimetric analysis

The isobaric heat capacity (C_p) and thermogravimetry (TG) measurements of samples were carried out using a Netzsch STA449 F1 instrument under an Ar atmosphere. The samples were put in a platinum crucible situated on a sample holder of the DSC at room temperature. Then, the samples were heated at 10 K min^{-1} to the target temperature. After cooling to room temperature, the second upscan was performed using the same procedure. To determine the C_p of the samples, both the baseline (blank) and the reference sample (sapphire) were measured.

Measurements of magnetic properties

The magnetic properties of the as-synthesized Fe-ZIF, Fe-ZIF-593K and Fe-ZIF glass samples

were characterized by using Quantum Design MPMS3 and LakeShore 7404. The temperature-dependent magnetization (*M-T*) curves were recorded over two temperature ranges: 323-800 K at 100 Oe and 2-400 K at 1000 Oe, upon heating rate of 10 K min^{-1}. The magnetization versus field-dependent (*M-H*) curves were collected at room temperature, under the magnetic field up to ± 20000 Oe.

X-ray diffraction

Room-temperature XRD data (2θ = 5° to 50°) were collected using a Bruker D8 advance X-ray powder diffractometer (Cu Kα).

Scanning electron microscope

SEM images of samples were taken using a Zeiss sigma 500. Prior to observations, all samples were coated with Pt for 120 s in a vacuum to increase their conductivity.

Optical microscope

Optical microscope photographs of samples were recorded by using Olympus CX33.

Differential scanning calorimetry - Thermogravimetric analysis - Mass spectrometry

DSC-TG-MS analysis was conducted using simultaneous TG-DSC instrumentation coupled with a mass spectrometer. Specifically, a NETZSCH STA 449 F3 Jupiter was integrated with a NETZSCH QMS 403 D Aeolos. The same procedures were identical to those employed in the DSC analysis, with the subsequent characterization of the released volatiles performed via mass spectrometry.

FT-Raman spectroscopy

Raman spectra were recorded using a Thermo Scientific IS50 Micro-Raman spectrometer equipped with a neodymium-doped yttrium aluminium garnet (Nd:YAG) laser (1064 nm). The spectral resolution was 4 cm^{-1}. A typical power of 2.4 mW was used to record spectra in the range of 100 to 2000 cm^{-1}.

Mössbauer spectroscopy

The ^{57}Fe Mössbauer spectra of samples were obtained at 300 K using a Wissel MR-2500

Mössbauer spectrometer with a ^{57}Co/Rh source in transmission geometry over the velocity range of ± 12.5 mm s^{-1} under high vacuum for approximately 48 h. The data were fitted using the MossWinn 4.0 software.

X-ray photoelectron spectroscopy

The XPS spectra of samples were collected using a Thermo Scientific K-Alpha instrument equipped with a monochromatic Al Kα X-ray source (hv = 1486.6 eV).

High-energy synchrotron X-ray diffraction

High-energy synchrotron X-ray diffraction (HEXRD) were conducted at Beamline 12SW at the Shanghai synchrotron radiation facility (SSRF). Focused high-energy X-ray beam (0.5mm × 0.5 mm) with wavelength 0.13918 Å (89.0794 keV) was employed in this work. Fine powders were sealed in a Kapton capillary with diameter 1.25 mm. The filled capillary was mounted on an aluminum alloy frame using Kapton tapes, and the diffraction signals were collected by a large-area detector (Pilatus 2M, 1475 × 1679 pixels of 172 μm × 172 μm) placed 319.134 mm downstream of the sample. This sample-to-detector distance was calibrated using the standard diffraction pattern from polycrystalline CeO_2 powder. The measurement procedure was controlled by an in-house software and total 120 s exposure time (divided into 4 independent patterns) was set for all of the samples. Background patterns were acquired with the same set up and exposure strategy.

These 2D diffraction patterns collected in HEXRD were converted into Q-Intensity curves using *FIT2D* program, with wavevector Q is calculated as Q=4π sin(2θ)/λ (2θ is the scattering angle and λ is the X-ray wavelength). The resulting data was further corrected for polarization and sample attenuation to obtain the scandalized intensity $I(Q)$, and then the total structural factors $S(Q)$ can be extracted. In this work, the calculated $S(Q)$ patterns covered the Q range of 0.5-20.5 Å^{-1}, which was used to derive the reduced pair distribution functions (PDF) $G(r)$ in real space by inverse Fourier transformation with Lorch function $L(Q)$ as window function.[1]

Calculation Details

In this study, we perform density functional theories (DFT) to study on the electronic structure. We employed the plan wave basis to expand the wave functions,[2] and the exchange-correlation energy functional was deal with generalized-gradient approximation (GGA).[3-5] Specifically, the Perdew-Burker-Ernzerhof (PBE) exchange-functional was adopted.[6,7] Each self-consistent electronic calculation is converged to within 10^{-8} Ry, the ionic and cell relaxation is iterated until the forces are less than 10^{-5} Ry/bohr. In our calculations, the *k*-resolved set to 4×4×4, the plan-wave basis of the kinetic and charge density was extended up to cut-off energy of 45 Ry and 450 Ry. The codes used for the present calculations are freely available with the QUANTUM-ESPRESSO (QE) package v7.4.[8] Wannier90 calculations were performed ensuring a correct fit the electronic band structure and spreads.[9] Non-collinear TB2J calculations were performed with a 2×2×2 supercell and carefully selected limits of the exchange integral.[10] More calculations are provided in Table S1.

Supplementary Figures

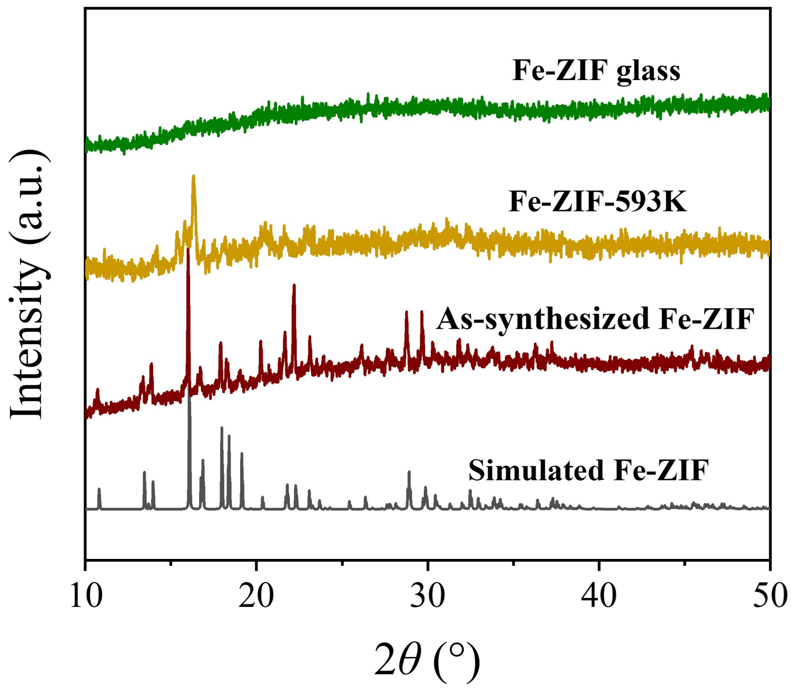

Figure S1. XRD patterns recorded at room temperature for as-synthesized Fe-ZIF, Fe-ZIF-593K and Fe-ZIF glass.

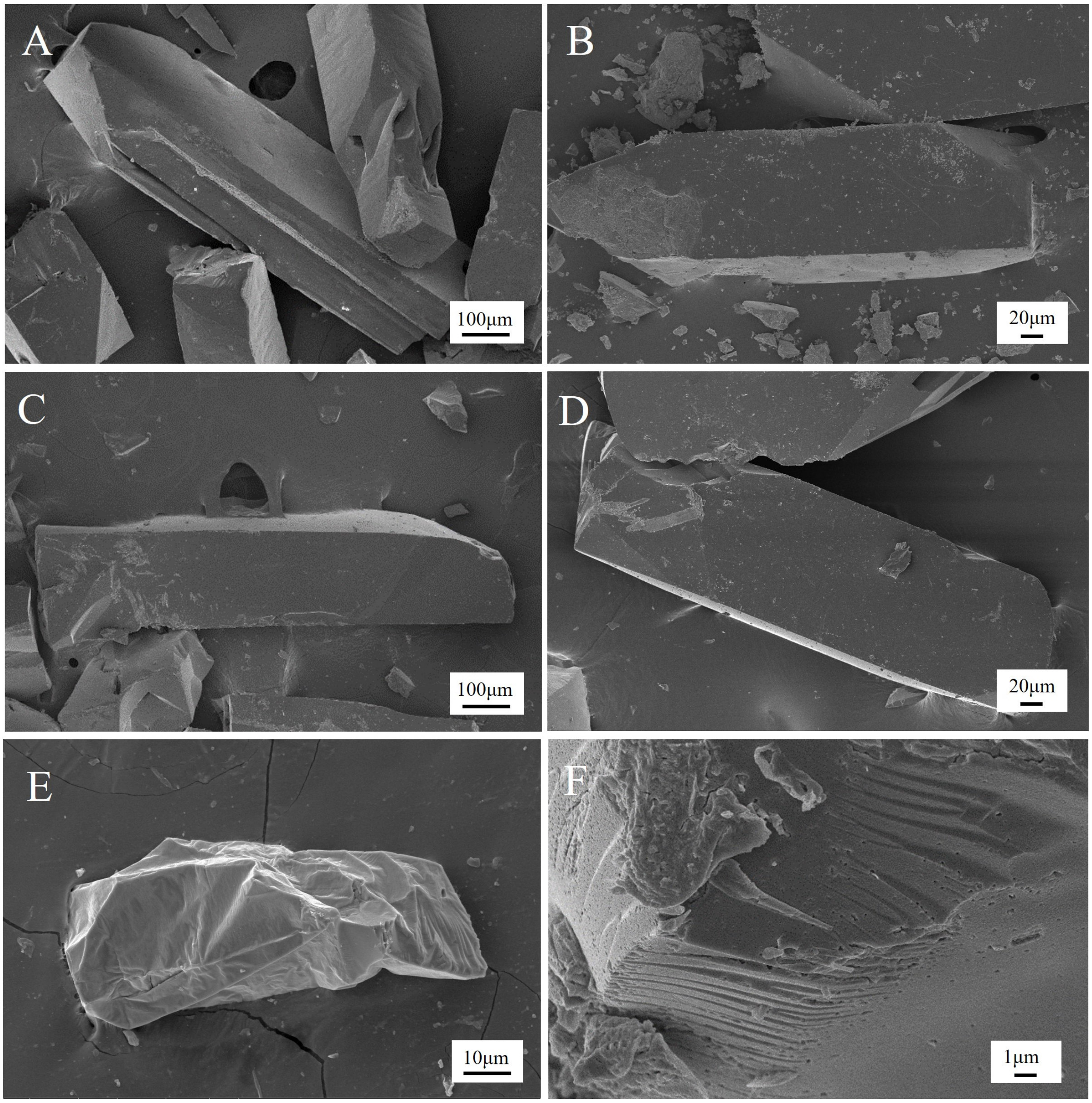

Figure S2. SEM images of the as-synthesized Fe-ZIF (A-B), Fe-ZIF-593K (C-D), and Fe-ZIF glass (E-F).

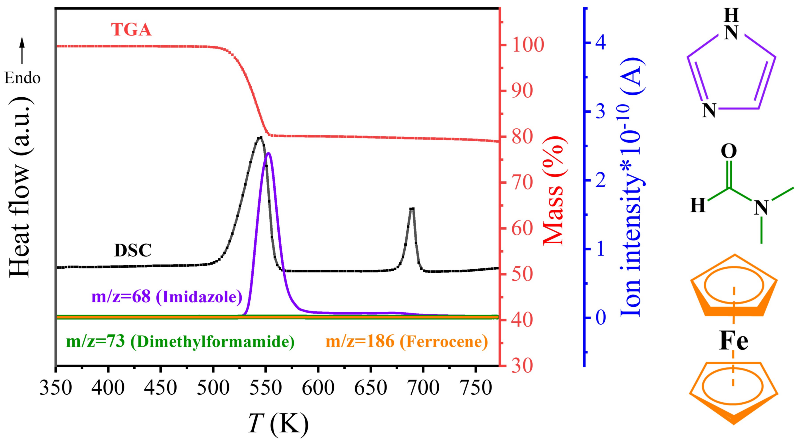

Figure S3. TG-DSC-MS study of as-synthesized Fe-ZIF. The red line corresponds with the TGA analysis. It was observed that significant fluctuations in ion intensity occurred exclusively at m/z=68, which is associated with imidazole.

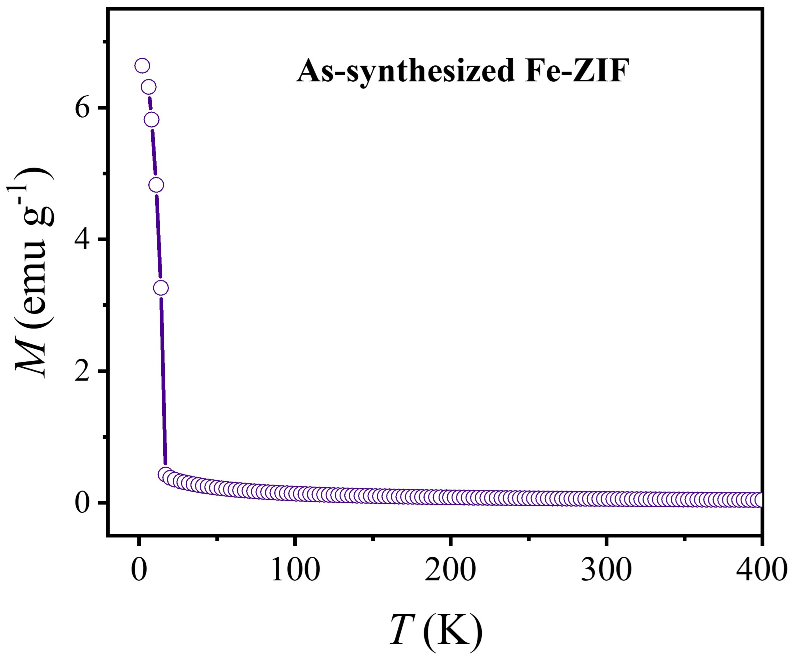

Figure S4. Magnetization versus temperature (*M*-*T*) curve of as-synthesized Fe-ZIF, recorded at 1000 Oe from 2-400 K.

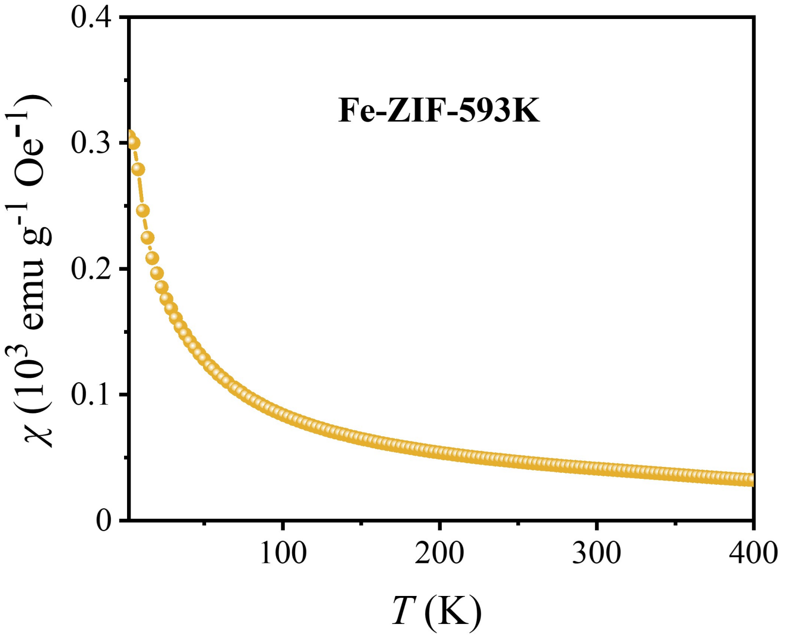

Figure S5. Temperature dependence of magnetic susceptibility (χ-T) curves of Fe-ZIF-593K sample.

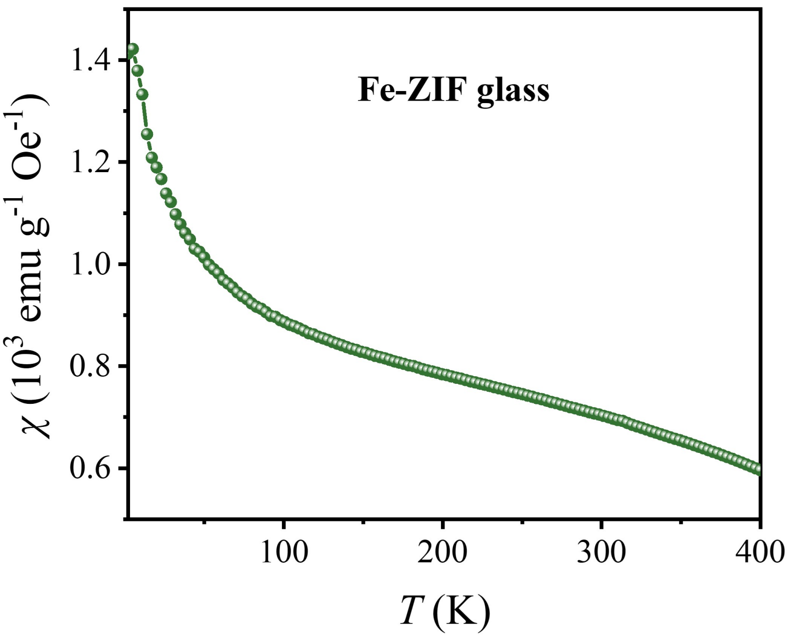

Figure S6. Temperature dependence of magnetic susceptibility (χ-T) curves of Fe-ZIF glass sample.

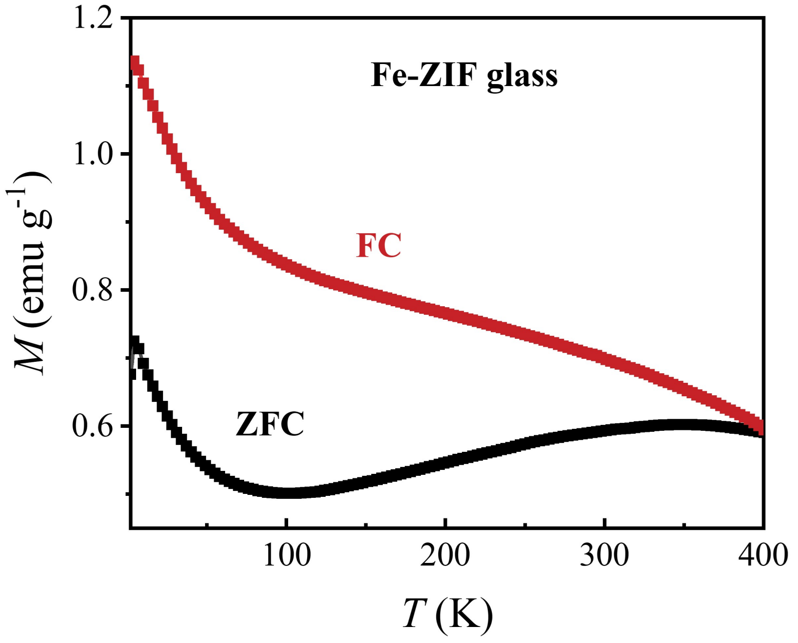

Figure S7. Zero-field cooled (ZFC) and field cooled (FC) curves of Fe-ZIF glass under an applied field of 1000 Oe.

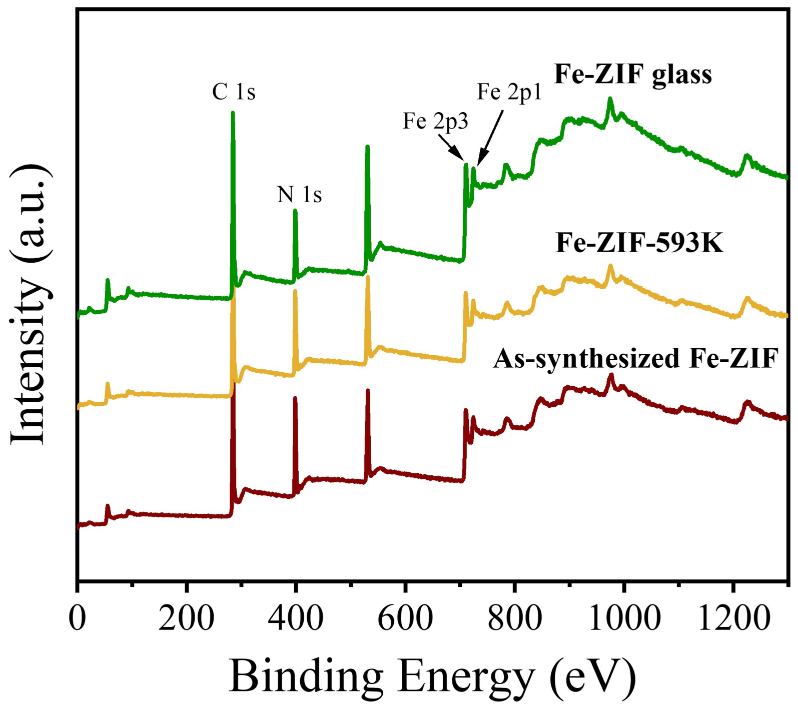

Figure S8. Survey XPS spectra of as-synthesized Fe-ZIF, Fe-ZIF-593K and Fe-ZIF glass.

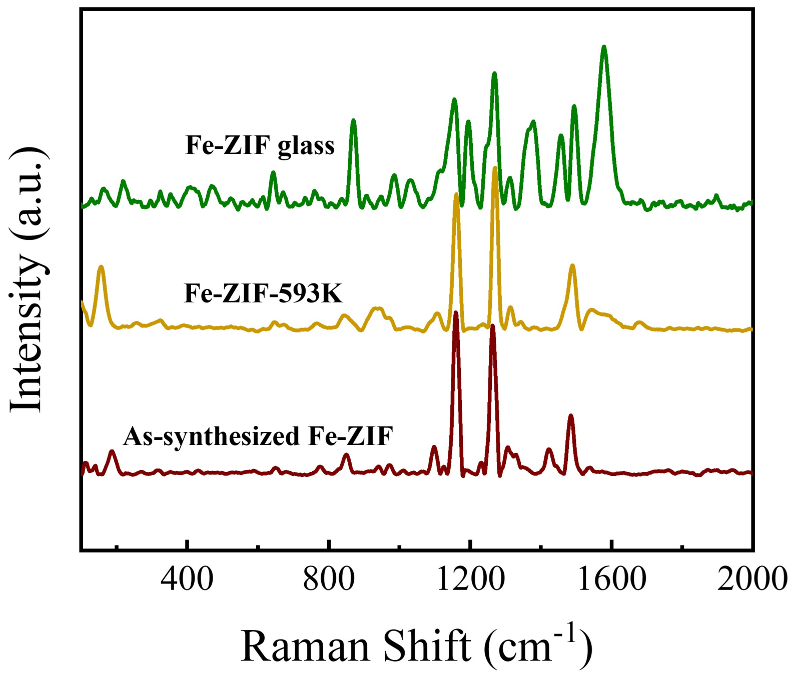

Figure S9. Normalized FT-Raman spectra recorded at the region of 100-2000 cm^{-1} of as-synthesized Fe-ZIF, Fe-ZIF-593 K and Fe-ZIF glass.

Supplementary Tables

Table S1. Calculated Magnetic moments, exchange coupling constants and their spin-interactions in the as-synthesized Fe-ZIF. Inter-atomic distance corresponding to each-interaction is also given, Fe_1, Fe_2 and Fe_3, respectively.

Bond	Inter-atomic distance (Å)	Coupling constant (meV)	Nature of spin-interaction
Fe_1 - Fe_2	6.14	-1.5334	AFM
Fe_2 - Fe_3	6.21	-0.4040	AFM
Fe_3 - Fe_1	7.35	-0.0071	AFM

Table S2. Raman peaks assignments.

Samples / Assignment	Fe-ZIF	Fe-ZIF -593K	Fe-ZIF glass
FeN_6 HS[11]	108	--	--
δ (N-Fe-N) LS[12]	--	158	--
v (Fe-N) HS[13,14]	--	--	220
v (C-N)[15]	1160	1160	1153
v (C-N)[15]	--	--	1194
v (Im) breathing+ *β*(C-H)[16]	1262	1270	1268
w (CH_2)[17]	--	--	1375
w (CH)[17]	--	--	1456
v (CN) + *w* (N-H)[17]	--	--	1496

Abbreviations: HS: high spin, HS: low spin, *v*: stretching, *w*: wagging, *β*: in-plane deformation, *δ*: bending.

Table S3. Mössbauer parameters derived from the fittings. Isomer shift (IS), quadrupole splitting (QS) and relative spectral area % of samples.

Sample	IS ($mm\ s^{-1}$)	QS ($mm\ s^{-1}$)	Area (%)	Assignment
As-synthesized Fe-ZIF	1.24	1.72	35.6	$Fe-N_6$ (Fe^{II} HS)
	0.72	2.29	64.4	$Fe-N_4$ (Fe^{II} MS)
Fe-ZIF-593 K	0.22	1.48	60.5	D1 [$Fe-N_4$ (Fe^{II} LS)]
	0.26	0.79	39.5	D2 [$Fe-N_4$ (Fe^{II} LS)]
Fe-ZIF glass	0.80	2.30	100	$Fe-N_4$ (Fe^{II} HS)

References

[1] E. Lorch, *J. Phys. C: Solid State Phys.* **1969**, *2*, 229.

[2] P. E. Blöchl, *Phys. Rev. B.* **1994**, *50*, 17953-17979.

[3] G. Kresse, J. Furthmüller, *Phys. Rev. B.* **1996**, *54*, 11169-11186.

[4] G. Kresse, D. Joubert, *Phys. Rev. B.* **1999**, *59*, 1758-1775.

[5] J. P. Perdew, K. Burke, M. Ernzerhof, *Phys. Rev. Lett.* **1996**, *77*, 3865-3868.

[6] J. Klimeš, D. R. Bowler, A. Michaelides, *J Phys-condens Mat.* **2010**, *22*, 022201.

[7] M. Gajdoš, K. Hummer, G. Kresse, J. Furthmüller, F. Bechstedt, *Phys. Rev. B.* **2006**, *73*, 045112.

[8] P. Giannozzi, S. Baroni, N. Bonini, M. Calandra, R. Car, C. Cavazzoni, D. Ceresoli, G. L. Chiarotti, M. Cococcioni, I. Dabo, A. Dal Corso, S. de Gironcoli, S. Fabris, G. Fratesi, R. Gebauer, U. Gerstmann, C. Gougoussis, A. Kokalj, M. Lazzeri, L. Martin-Samos, N. Marzari, F. Mauri, R. Mazzarello, S. Paolini, A. Pasquarello, L. Paulatto, C. Sbraccia, S. Scandolo, G. Sclauzero, A. P. Seitsonen, A. Smogunov, P. Umari, R. M. Wentzcovitch, *J Phys-condens Mat.* **2009**, *21*, 395502.

[9] N. Marzari, D. Vanderbilt, *Phys. Rev. B.* **1997**, *56*, 12847-12865.

[10] H. Wang, J. Qi, X. Qian, *Appl. Phys. Lett.* **2020**, *117*.

[11] Z. G. Lada, K. S. Andrikopoulos, C. D. Polyzou, V. Tangoulis, G. A. Voyiatzis, *J. Raman Spectrosc.* **2020**, *51*, 2171-2181.

[12] Z. G. Lada, K. S. Andrikopoulos, A. Chrissanthopoulos, S. P. Perlepes, G. A. Voyiatzis, *Inorg. Chem.* **2019**, *58*, 5183-5195.

[13] E. Smit, B. Manoun, D. d. Waal, *J. Raman Spectrosc.* **2001**, *32*, 339-344.

[14] Y. Suffren, F.-G. Rollet, O. Levasseur-Grenon, C. Reber, *Polyhedron* **2013**, *52*, 1081-1089.

[15] L. Frentzel-Beyme, M. Kloß, R. Pallach, S. Salamon, H. Moldenhauer, J. Landers, H. Wende, J. Debus, S. Henke, *J. Mater. Chem. A.* **2019**, *7*, 985-990.

[16] A. Zdaniauskienė, M. Talaikis, T. Charkova, R. Sadzevičienė, L. Labanauskas, G. Niaura, *Molecules.* **2022**, *27*, 6531.

[17] G. Kumari, K. Jayaramulu, T. K. Maji, C. Narayana, *J. Phys. Chem. A.* **2013**, *117*, 11006-11012.
